# Supplementary material for: Optimizing Data for Modeling Neuronal Responses
Source: Front Neurosci. 2019 Jan 10;12:986. doi: 10.3389/fnins.2018.00986 (PMC6335328; doi:10.3389/fnins.2018.00986)
Supplement: Supplementary file 1 [file Data_Sheet_1.PDF]

Optimising data for modelling neuronal responses

Supplementary Material

Slice leakage / aliasing analysis

As with any accelerated imaging technique, the mulitband acquisition scheme is vulnerable to potential aliased signals not being unfolded correctly. This is important since activation aliasing between DCM regions of interest could potentially lead to artificial correlations between regions (Todd et al., 2016). Successful multiband (MB) reconstruction can be expected to produce the same percentage of false positive voxels in aliased and non-aliased voxels. To investigate this in our data, we created subject-specific masks corresponding to the aliased locations of all seven ROIs for a given MB factor. The ROIs were projected from normalized space back into native space using the inverse deformation field and the aliased locations for these voxels were then determined for MB 2, MB 4, and MB 8 based on their specific aliasing pattern (given MB factor and CAIPI-shift employed). These aliased locations were then projected back into normalized space using the forward deformation field. All seven ROIs therefore have [MB-1] aliased locations that are shifted diagonally along the slice and phase encode direction from the originally defined ROI. We first checked whether any of the DCM ROIs overlaped with an aliased region. Next, the following four measures were calculated: 1) α: the total number of activated voxels in the aliased regions, 2) β: the total number of voxels in the aliased regions, 3) δ: the total number of voxels activated elsewhere in the brain (i.e. outside the ROIs used in the DCM analysis or their aliased locations) and, 4) γ: total number of voxels elsewhere in the brain. A Wilcoxon signed-rank test was performed to test the hypothesis that the percentage of activated voxels in the alised regions (α/β) was not significantly higher than the percentage of voxels activated elsewhere (δ/γ).

For each MB factor considered, the aliased location of a given ROI used in the DCM analysis did not overlap with any other ROI being used in the analysis. In addition, the number of activated voxels in the aliased locations of the ROIs used in the DCM analysis was not significantly higher than in non-aliased locations outside the ROIs under consideration (Wilcoxon signed-rank paired test results were: p=0.2783 for MB=2, p=1 for MB=4 and p=0.9678 for MB8). In the case of MB=2, the peak number of aliased voxels was 81 corresponding to 6.8% in one subject (with an average of 2.4% across all ten subjects). The peak number of aliased voxels for MB=4 was 83 corresponding to 2.9 % in one subject (with an average of 0.9 % across all ten subjects). The peak number of aliased voxels for MB=8 was 77 corresponding to 2.5% in one subject (with overall average 0.6% across all ten subjects).

References

Todd N, Moeller S, Auerbach EJ, Yacoub E, Flandin G, Weiskopf N (2016) Evaluation of 2D multiband EPI imaging for high-resolution, whole-brain, task-based fMRI studies at 3T: Sensitivity and slice leakage artifacts. NeuroImage 124:32-42.
